# Dating violence perpetration and victimization among high schoolers from public and private schools in Rio de Janeiro, Brazil

**DOI:** 10.1590/0102-311XEN149923

**Published:** 2025-02-07

**Authors:** Claudia Leite de Moraes, Daniela Porto Faus, Márcia C. Castro, Michael Reichenheim, Washington Leite Junger, Stella Regina Taquette

**Affiliations:** 1 Instituto de Medicina Social Hesio Cordeiro, Universidade do Estado do Rio de Janeiro, Rio de Janeiro, Brasil.; 2 Programa de Pós-graduação em Saúde da Família, Universidade Estácio de Sá, Rio de Janeiro, Brasil.; 3 Universidade Federal do Rio de Janeiro, Rio de Janeiro, Brasil.; 4 Harvard School of Public Health, Boston, U.S.A.; 5 Universidade do Estado do Rio de Janeiro, Rio de Janeiro, Brasil.

**Keywords:** Intimate Partner Violence, Child Abuse, Adolescent, Violência por Parceiro Íntimo, Maus-tratos Infantis, Adolescente, Violencia de Pareja, Maltrato a los Niños, Adolescente

## Abstract

Dating violence during adolescence is a global public health issue due to its widespread occurrence and negative health consequences. Unfortunately, research addressing this topic in Latin American countries remains limited. Seeking to bridge this gap, the present study estimated the prevalence of both dating violence victimization and perpetration among high schoolers considering sociodemographic factors, exposure to other forms of violence, and school-related characteristics. A total of 539 students participated in the study selected by means of probabilistic sampling from private and public schools in Rio de Janeiro, Brazil. Dating violence was identified using the *Conflict in Adolescent Dating Relationships Inventory*. Victimization prevalence ranged from 16.7% (sexual) to 94.6% (emotional), whereas perpetration prevalence varied between 9.9% (sexual) and 94.6% (emotional). Boys were more physically victimized (37.2% vs. 24.5%) and perpetrated more sexual abuse (13.7% vs. 6.9%) than girls. Adolescents reporting child abuse, living in violent areas, and those who consumed alcoholic beverages more frequently had greater dating violence prevalence overall. However, some differences between male and female vulnerability characteristics merit debate. These heterogeneous profiles of victimization/perpetration between boys and girls indicate the need for specific dating violence prevention interventions.

## Abstract (pt)

A violência no namoro durante a adolescência é um problema de saúde pública mundial devido à sua ocorrência e consequências negativas para a saúde. Infelizmente, as pesquisas que abordam essa questão nos países latino-americanos ainda são limitadas. Buscando preencher essa lacuna, este estudo busca estimar a prevalência de vitimização e perpetração de violência no namoro entre estudantes do Ensino Médio, considerando fatores sociodemográficos, exposição a outras formas de violência e características relacionadas à escola. A pesquisa inclui 539 participantes selecionados por um procedimento de amostragem probabilística de escolas públicas e privadas do Rio de Janeiro, Brasil. O *Conflict in Adolescent Dating Relationships Inventory* foi utilizado para detectar violência no namoro. A prevalência de vitimização variou de 16,7% (sexual) a 94,6% (emocional). A prevalência de perpetração variou entre 9,9% (sexual) e 94,6% (emocional). Os meninos foram mais vitimizados fisicamente (37,2% *vs*. 24,5%) e perpetraram mais abuso sexual (13,7% *vs*. 6,9%) do que as meninas. Em geral, os adolescentes que relataram abuso infantil, os que vivem em áreas violentas e os que consomem bebidas alcoólicas com maior frequência apresentaram maior prevalência de violência no namoro. No entanto, algumas diferenças entre as características de vulnerabilidade de meninos e meninas precisam ser mais bem debatidas. Esses perfis heterogêneos de vitimização/perpetração de meninos e meninas indicam a necessidade de intervenções específicas para prevenir a violência no namoro.

## Abstract (es)

La violencia en el noviazgo durante la adolescencia es un problema de salud pública mundial debido a su aparición y sus consecuencias negativas para la salud. Desafortunadamente, las investigaciones que abordan este tema en los países latinoamericanos aún son limitadas. Para tratar de llenar este vacío, este estudio busca estimar la prevalencia de victimización y perpetración de violencia en el noviazgo entre estudiantes de secundaria, teniendo en cuenta factores sociodemográficos, exposición a otras formas de violencia y características relacionadas con la escuela. La investigación incluye 539 participantes seleccionados mediante un procedimiento de muestreo probabilístico de escuelas públicas y privadas de Río de Janeiro, Brasil. Se utilizó el *Conflict in Adolescent Dating Relationships Inventory* para detectar violencia en el noviazgo. La prevalencia de victimización osciló entre el 16,7% (sexual) y el 94,6% (emocional). La prevalencia de perpetración osciló entre el 9,9% (sexual) y el 94,6% (emocional). Los niños fueron más victimizados físicamente (37,2% *vs*. 24,5%) y perpetraron más abusos sexuales (13,7% *vs*. 6,9%) que las niñas. En general, los adolescentes que reportaron maltrato infantil, los que viven en zonas violentas y los que beben alcohol con mayor frecuencia presentaron mayor prevalencia de violencia en el noviazgo. Sin embargo, es necesario analizar más a fondo algunas diferencias entre las características de vulnerabilidad de niños y niñas. Estos perfiles heterogéneos de victimización/perpetración de niños y niñas indican la necesidad de intervenciones específicas para prevenir la violencia en el noviazgo.

## Introduction

The U.S. Centers for Disease Control and Prevention (CDC) ^1^ defines dating violence as situations involving physical, sexual, and psychological aggression or stalking in dating or occasional affective-sexual relations which may occur personally or electronically, between current or past partners, whether involving a heterosexual or homosexual relationship. Recent meta-analysis including studies from different countries estimated that 20% and 9% of adolescents engaged, respectively, in physical and sexual violence in the 12 months preceding the interviews ^2^. According to the study, girls perpetrate more physical aggression than boys (25% vs. 13%); however, reports from respondents as victims showed no difference between genders (21% in boys and girls). A different profile was observed for sexual violence, with girls reporting lower perpetration (3% vs. 10%) yet a higher victimization prevalence (14% vs. 8%).

A relatively recent review including 12 studies found victimization rates of cyber dating violence ranging from 12% to 56%, and perpetration rates between 12% and 54% ^3^. Despite research indicating that dating violence can be bidirectional, studies suggest that girls tend to suffer more severe psychological and physical harm and are primarily the victims of sexual violence ^4^ ^,^ ^5^ ^,^ ^6^. But comprehensive global data on this issue remains lacking ^7^. Failure to use questionnaires specifically built to capture intimate partner violence in this life cycle emerged as a relevant limitation in most of the studies. The review also highlighted that most studies come from high-income nations albeit the issue is more frequent in low- and middle-income countries (LMICs) ^2^. Despite the growing literature on this topic, the number of studies is still reduced when compared with those focusing on adulthood.

In Brazil, as in other Latin American countries, relatively few studies have quantified the problem among adolescents. The first survey was conducted in 2008 with 3,205 students between 15 and 19 years of age, covering ten state capitals and the Federal District. Study findings reported that 30% of the participants had suffered emotional violence, 24.2% were victims of dating threats, and 29.2% had perpetrated this type of violence in their last intimate relationship ^8^. Physical violence involved 24.1% of the adolescents as perpetrators and 19.6% reported victimization ^9^. A more geographically restricted survey on public schools in the state of Pernambuco draws attention to the high prevalence of physical dating violence and the differences in perpetration and victimization estimates between boys and girls. Girls claimed a higher frequency of perpetration (14.3% vs. 7.6%), whereas boys reported higher victimization rates (18.5% vs. 3%) ^10^. Similar differences according to genders was found in a sample of 428 adolescents characterized as perpetrators from the metropolitan region of Porto Alegre (Rio Grande do Sul State) ^11^. However, dating violence severity seems to be higher among girls, as suggested by another Brazilian paper ^12^.

Global thematic literature has been indicating that dating violence have social, contextual, relational, and individual determinants ^6^ ^,^ ^13^ ^,^ ^14^ ^,^ ^15^; however, few Brazilian epidemiological studies have investigated how dating violence prevalence changes according to these dimensions ^9^ ^,^ ^16^ ^,^ ^17^. Such study ^9^ has examined this topic with a sufficiently large sample to identify prevalence differences among population subgroups. Specifically, in terms of correlations between exposure to violence during childhood and adolescence, only one study has explored the topic ^18^, whereas none have investigated the distribution of dating violence prevalence according to community victimization history.

Such scarcity of studies focusing on dating violence among adolescents has commonly led to extrapolating data from intimate partner violence research on adults ^19^, which is of concern since intimate relationship profiles may differ in these two periods of life. Emotional immaturity specific to younger age groups coupled with the fact that adolescents are still developing their social skills and building their own identity impart distinctive characteristics to aggression during adolescence ^13^. As they are still learning how to conduct themselves in partner relationships, adolescents may find it challenging to cope with conflicts without using violent and coercive acts for its resolution. In turn, as they often live with their parents, adolescents spend less time with partners and are thus less physically exposed ^13^. Cyberbullying and revenge pornography are also more common among adolescents than between adult couples ^20^.

Intimate partner violence-related negative consequences during adolescence also bear particular features and reinforce its relevance. Injuries and traumas resulting from physical abuse, sexually transmitted infections, risky sexual behavior, and unwanted pregnancies are notably more frequent among this population ^4^. Mental health issues are also more common and harmful ^21^. Additionally, recognition that dating violence early in life is a substantial risk factor for intimate partner violence in adulthood reinforces the need for more studies focusing on adolescents and youth ^13^.

Gathering knowledge on the magnitude and characteristics of the situations occurring among adolescents is a first step for increasing its visibility and raising awareness of the need to confront it. In this regard, this paper estimated the victimization and perpetration prevalence of emotional, physical, sexual, and threatening behavior among high schoolers from Rio de Janeiro, Brazil. As a secondary objective, we examined whether prevalence varies according to sociodemographic and school characteristics, child abuse reports, exposure to community violence, and alcohol consumption.

## Methods

### Study setting

This research involved second-year high schoolers from public and private schools located in the IX Administrative Region (IX-AR) of Rio de Janeiro city. IX-AR comprises four neighborhoods with approximately 190,000 inhabitants, according to the 2010 Brazilian census ^22^, and a monthly average per capita income of 1836 BRL, which corresponded to USD 1,020 at the time ^23^. Despite having the fifth-highest Human Development Index (HDI) in the city, the region shows a striking inequality pattern. Middle- and upper-class houses, buildings, and condos are surrounded by slums and low-income communities (e.g., Borel, Macacos, and Complexo do Andaraí) marked by lack of sanitation, precarious housing conditions, and high levels of violence ^24^.

### Participants

Source population consisted of all students regularly enrolled in the 2^nd^ year (high school) in the IX-AR of Rio de Janeiro in 2016. This totaled 1,470 students across 52 classes, distributed among five public schools with both daytime and night classes and 15 private schools offering only daytime classes. Sample size calculation was based on the study’s main focus on exploring prevalence by gender and the use of logistic regression models with binary exposure variables and covariates adjustments. Calculation first considered a significance level (alpha) of 0.05, a 25% prevalence of physical violence, and a 0.05 margin of error as parameters. Assumptions also included a 15% non-response rate and full population coverage. As stratification focused on gender domains, we doubled the sample size, anticipating 678 students (46.1% of the sample fraction) for prevalence estimations. Subsequently, fine-tuning was performed to accommodate multivariable analysis exploring associations between dating violence and specific health outcomes (e.g., substance abuse, common mental disorders, and early sexual initiation), projecting a sample size of 726 adolescents (49.4% of the sample fraction).

Students were selected by cluster sampling stratified according to three groups: private schools with daytime classes, public schools with daytime classes, and public schools with night classes. Considering the average number of students per class, we chose 26 classes with a selection probability proportional to school size. All students from the selected classes who were regularly attending school were invited to participate. Of the 747 eligible students, 721 agreed to participate in the research (95.4%). Among these, 556 reported having a dating partner within a 12-month recall period, making them eligible for the present study. Due to the small number of self-reported Indigenous (n = 12) and Asian (n = 5) and students, and supposing that if included in the analysis they would generate a rather imprecise subgroup estimate, we decided to exclude them from the study. Thus, the final study sample comprised 539 students.

### Fieldwork and measurements

Fieldwork involved prior contact with schools, distribution and collection of signed Informed Assent and Consent Forms for adolescents and their families, student sensitization in the week preceding data collection, and the actual data collection. Information was obtained using a multidimensional self-completion questionnaire applied in class. Data collection took place between September 2016 and February 2017. Dating violence was identified by the Portuguese version of the *Conflict in Adolescent Dating Relationships Inventory* (CADRI) ^9^ ^,^ ^25^, a self-report questionnaire developed in Canada focusing on detecting different forms of dating violence among adolescents. The instrument consists of 25 items distributed in five subscales: physical violence (4 items), emotional violence (10), sexual violence (4), threatening behavior (4), and relational violence (3 items). Items hold four level responses (often, sometimes, seldom, and never), and cover a 12-month recall period. A recent review of CADRI’s psychometric history identified studies evaluating its internal consistency ^9^ ^,^ ^25^ ^,^ ^26^ ^,^ ^27^ and an acceptable test-retest reliability ^9^ ^,^ ^25^ ^,^ ^26^. Positive correlations between CADRI and other instruments assessing trauma and anxiety support the validity of the instrument ^28^.

The present study used the physical, emotional, threatening behavior, and sexual violence subscales. The item referring to forced kissing was removed from the sexual subscale due to its low reliability in the Brazilian version ^29^. Participants answered as victims and perpetrators. Adolescents who answered positively at least one of the subscale items were considered positive for that form of violence.

Demographic variables included gender (man or woman), age in years (15 ≤ 16, 17, ≥ 18-19), and current religion (catholic, evangelical, spiritualist, *umbanda* and *candomblé*, others, without religion). Family economic status was measured using the 2015 Brazilian Economic Classification Criteria ^30^ which define broad economic classes based on factors such as ownership of durable goods, domestic employee contracts, schooling level of the family head, and access to public services. Using a scoring system that weighs the number of items present in the household and other relevant characteristics, families are classified into A, B, C, D, and E economic classes, with A having the highest and E the lowest purchasing power ^30^. Due to the small number of families in the extremes, the highest (A and B) were grouped into one category, and the lowest (C, D, and E) into another. Maternal education (≤ 5 years of schooling, 6-8 years of schooling, and > 8 years of schooling) and whether the student lives with both parents (yes or no) represent, respectively, the socioeconomic background and family structure. School type (private or public) and period (day or night) characterized the schools.

Childhood abuse (up to 10 years of age) was verified by recall using the Brazilian version of the *Childhood Trauma Questionnaire* (CTQ) ^31^ ^,^ ^32^, a 28-item scale comprising five subscales: emotional, physical, and sexual abuse, and physical and emotional neglect. Subscales comprise five items each since three items are only used to enhance the interview. Items have five alternatives: never, a few times, sometimes, many times and always. This study considered all options but never as positive for the item. A positive answer to at least one item was considered as positive reporting for the respective subscale.

Exposure to community violence was assessed by asking if the student had seen the body of someone murdered or if they ever had a relative, friend, neighbor, or colleague killed. Students’ involvement with youth violence was identified by asking if they had a colleague or friend who had done any of the following acts in the previous 12 months: (i) carried a firearm; (ii) threatened someone with a firearm; (iii) killed someone; (iv) threatened someone with death; (v) assaulted someone; (vi) was wounded by a firearm; (vii) wounded someone with a firearm; or (viii) beat someone ^33^.

Alcohol use frequency in the prior three months was assessed by the World Health Organization (WHO)-endorsed *Alcohol, Smoking, and Substance Involvement Screening Test* (ASSIST) questionnaire ^34^. Participants were grouped into three categories: never, sometimes (combining once or twice and once a month), and often (merging once a week and almost every day) ^34^.

### Data analysis

All data analyses accounted for the complex sampling design (sampling weights and clustering). Dating violence prevalence and respective 95% confidence intervals (95%CI) were estimated for the total sample. Heterogeneity of proportions in population subgroups was evaluated by the chi-squared test, with an alpha < 0.05 to delimit statistically significant differences. All analyses were conducted in R software, version 3.6.1 (http://www.r-project.org). Sample weights were incorporated using the *survey* package.

### Ethics

The research protocol was approved by the Research Ethics Committee of the State University of Rio de Janeiro (Certificate of Presentation for Ethical Appreciation n. 48107514.2.0000.5282), and by the Rio de Janeiro State Department of Education.

## Results

Our study sample comprised adolescents aged between 15 and 19, averaging 17 (standard deviation - SD = 0.93) years old. Table 1 shows a slight prevalence of students auto-declared female. Approximately 70% stated affiliation to some religion, nearly 50% of those professing catholic or evangelic denominations. Over 80% of the total sample were from middle economic classes (classes B + C), and two-fifths of participants had mothers with five or fewer years of schooling. Nearly 60% did not live with both parents. Public school students accounted for 40% of the sample, with only 10% attending night classes. Most of the sample reported some child abuse and neglect, and almost 60% of the students had already seen a murdered body on the streets or had a family member killed. About 40% had friends engaged in youth violence and 80% reported consuming alcohol in the prior three months.

**Table 1:** Sociodemographic characteristics, childhood abuse, community violence, and alcohol consumption among adolescent students in Rio de Janeiro, Brazil.

Parameter	n	%	95%CI
Demographic profile			
Gender			
Female	297	55.7	55.6-55.7
Male	242	44.3	44.3-44.4
Age (years)			
15 ≤ 16	207	46.2	46.2-46.3
17	187	31.4	31.3-31.4
≥ 18-19	145	22.4	22.4-22.5
Current religion			
Catholic	146	28.8	28.7-28.8
Evangelical	145	23.1	23.0-23.1
Without religion	168	33.0	32.9-33.0
Spiritualist	27	5.5	5.5-5.5
*Umbanda* and *candomblé*	26	5.4	5.3-5.4
Others	24	4.4	4.3-4.4
Socioeconomic status			
Economic class			
A (wealthier families)	69	17.7	17.6-17.7
B	254	54.1	54.0-54.2
C	165	27.1	27.0-27.1
D-E (poorer families)	9	1.2	1.2-1.2
Maternal education (years of schooling)			
≤ 5	226	39.1	39.0-39.2
6-8	96	15.0	15.0-15.1
> 8	190	45.9	45.8-46.0
Family structure			
Lives with both parents			
No	312	57.0	57.0-57.1
Yes	224	43.0	43.0-43.0
School characteristics			
School type			
Private	228	60.4	60.4-60.5
Public	311	39.6	39.5-39.6
Study period			
Day	450	89.6	89.6-89.7
Night	89	10.4	10.3-10.4
Childhood abuse			
Emotional			
No	114	19.5	19.5-19.6
Yes	418	80.5	80.4-80.5
Physical			
No	230	44.2	44.2-44.3
Yes	302	55.8	55.7-55.8
Sexual			
No	486	89.4	89.4-89.5
Yes	50	10.6	10.5-10.6
Emotional neglect			
No	109	19.9	19.9-20.0
Yes	424	80.1	80.1-80.1
Physical neglect			
No	291	56.8	56.7-56.8
Yes	237	43.2	43.1-43.3
Community violence and alcohol consumption			
Community violence			
No	220	41.1	41.1-41.2
Yes	313	58.9	58.8-58.9
Has friends that commited criminal acts			
No	338	63.5	41.1-41.2
Yes	197	36.5	58.8-58.9
Alcohol consumption in the last three months			
Never	129	21.9	21.9-21.9
Sometimes	246	49.6	49.5-49.6
Often	160	28.5	28.5-28.6

95%CI: 95% confidence interval.

Note: estimates considering sample weights.

Table 2 presents the dating violence prevalence. Victimization prevalence in the aggregate sample ranged from 16.7% (95%CI: 13.1-20.3) (sexual) to 94.6% (95%CI: 92.6-96.6) (emotional), whereas perpetration varied between 9.9% (95%CI: 7.4-12.4) (sexual) and 94.6% (95%CI: 92.6-96.6) (emotional). Only physical violence showed a statistically significant difference between gender, with boys being most frequently victimized. Regarding perpetration, girls reported perpetrating more threatening behaviors and physical violence against their boyfriends. Conversely, boys perpetrated sexual violence more often than girls.

**Table 2:** Prevalence of dating violence victimization and perpetration in the total sample and stratified by gender among adolescent students from Rio de Janeiro, Brazil.

Variables	Total			Female			Male		
	n	%	95%CI		n	%	95%CI			n	%	95%CI		
Victimization												
Threatening behavior	137	26.6	22.8	30.3	73	24.3	18.9	29.6	64	29.5	23.8	35.2
Emotional violence	500	94.6	92.6	96.6	277	94.4	92.1	96.6	223	94.9	92.0	97.8
Physical violence	158	30.1	26.2	33.9	75	24.5	18.1	30.9	83	37.2	31.5	42.9
Sexual violence	90	16.7	13.1	20.3	52	17.4	11.8	23.0	38	15.7	10.6	20.9
Perpetration												
Threatening behavior	159	27.5	22.6	32.4	105	32.35	24.9	39.8	54	21.4	16.9	25.8
Emotional violence	500	94.6	92.6	96.6	283	96.51	94.6	98.4	217	92.2	88.8	95.7
Physical violence	168	30.3	24. 7	35.9	119	37.56	28. 8	46.3	49	20.8	15.2	26.4
Sexual violence	58	9.9	7.4	12.4	23	6.86	3.9	9.8	35	13.7	9. 8	17.6

95%CI: confidence interval.

Note: estimates considering sample weights.

As shown in Table 3, victimization prevalence did not differ according to demographic, socioeconomic, family, and school-related factors in either gender, except for girls from families with higher economic status who declared themselves catholic or evangelic and boys not living with both parents. Emotional victimization was more often reported in these groups. However, victimization prevalence seems to be higher among adolescent reporting child abuse. Threatening behavior and physical violence were higher among girls who suffered childhood emotional abuse. Suffering threats was more frequent among boys who reported being victims of physical negligence, whereas emotional victimization was higher among those reporting childhood physical abuse. Sexual victimization was more frequent among boys and girls who suffered physical neglect and boys who reported a history of childhood emotional abuse. Prevalence of threatening dating behavior and physical victimization were also higher among those living in areas with higher levels of community violence. As for alcohol consumption, boys who drank alcohol more frequently in the prior three months showed a higher prevalence of threatening behavior and physical victimization. These situations do not vary according to alcohol consumption among girls.

**Table 3:** Prevalence of dating violence victimization according to subgroups among adolescent students from Rio de Janeiro, Brazil.

Variables	Threatening behavior	Emotional violence	Physical violence	Sexual violence	Threatening behavior	Emotional violence	Physical violence	Sexual violence
% (95%CI)	% (95%CI)	% (95%CI)	% (95%CI)	% (95%CI)	% (95%CI)	% (95%CI)	% (95%CI)
Demographic profile								
Age (years)								
15 ≤ 16	22.7 (15.7-29.7)	93.7 (89.1-98.3)	23.2 (11.6-34.7)	18.3 (9.4-27.3)	27.9 (19.4-36.4)	95.3 (90.2-100.0)	38.3 (29.0-47.6)	16.2 (7.7-24.7)
17	22.5 (14.8-30.3)	95.1 (90.1-99.6)	25.4 (18.7-32.1)	17.2 (10.8-23.7)	32.3 (18.7-45.8)	92.5 (86.2-98.8)	34.0 (21.1-46.9)	13.4 (5.3-21.4)
≥ 18-19	32.1 (19.6-44.5)	95.2 (90.8-99.7)	26.8 (14.0-39.6)	15.1 (4.9-25.3)	28.6 (17.8-39.5)	97.1 (93.9-100.0)	39.4 (22.6-56.5)	17.9 (7.9-27.8)
Current religion								
Catholic	21.0 (12.9-29.0)	98.3 * (96.0-100.0)	22.2 (8.9-35.6)	14.9 (7.2-22.6)	27.9 (12.0-43.9)	94.8 (89.5-100.0)	33.2 (17.8-48.5)	18.1 (7.5-28.8)
Evangelical	24.4 (13.6-35.1)	98.3 * (96.0-100.0)	23.7 (12.7-34.7)	15.9 (6.9-24.8)	41.3 (26.6-56.0)	96.9 (93.3-100.0)	48.3 (33.9-62.7)	14.2 (4.5-23.9)
Others **	26.8 (10.4-43.3)	88.3 * (80.2-96.3)	25.1 (13.5-36.7)	21.7 (9.0-34.4)	17.3 (0.0-39.4)	90.3 (79.6-100.0)	29.8 (10.4-49.1)	13.1 (0.0-28.6)
Without religion	25.0 (10.9-39.1)	92.1 * (86.0-98.2)	26.2 (15.5-36.9)	18.1 (7.5-28.7)	26.1 (17.0-35.1)	94.6 (90.3-98.9)	34.5 (26.9-42.0)	15.5 (7.6-23.3)
Socioeconomic status								
Economic status								
A-B (wealthier)	23.8 (16.0-31.5)	96.1 * (94.2-98.0)	23.9 (15.9-31.9)	18.0 (10.6 -25.5)	32.2 (24.7-39.7)	95.1 (91.7-98.5)	40.3 (33.2-47.4)	13.8 (7.2-20.3)
C-D-E (poorer)	25.0 (13.6-36.5)	91.7 * (86.2-97.1)	23.1 (15.5-30.8)	12.9 (6.6-19.3)	28.0 (15.6-40.3)	93.9 (89.0-98.8)	33.0 (21.1-45.0)	18.7 (9.8-27.6)
Maternal education (years of schooling)								
≤ 5	22.8 (13.6-32.0)	93.0 (88.1-98.0)	27.7 (18.3-37.2)	15.4 (7.7-23.0)	33.7 (25.4-42.0)	95.0 (90.8-99.1)	36.3 (27.4-45.1)	14.2 (6.6-21.7)
6-8	23.6 (15.2-32.0)	95.2 (92.2-98.2)	26.5 (15.7-38.0)	19.0 (9.3-28.7)	28.7 (19.6-37.9)	94.8 (90.3-99.2)	37.2 (27.6-46.8)	13.8 (5.0-22.6)
> 8	28.7 (11.1-46.3)	93.5 (86.7-100.0)	12.6 (5.0-20.2)	17.3 (7.1-27.4)	27.7 (12.4-43.1)	93.9 (84.8-100.0)	40.9 (25.5-56.3)	26.3 (13.2-39.4)
Family structure								
Live with both parents								
No	27.1 (19.5-34.7)	95.1 (91.2-99.0)	25.8 (17.1-34.4)	16.3 (9.9-22.7)	29.6 (22.0-37.3)	96.7 *** (94.1-99.4)	36.1 (28.1-44.2)	15.5 (10.2-20.7)
Yes	21.3 (12.6-30.0)	93.4 (89.4-97.5)	23.4 (15.1-30.8)	19.1 (11.7-26.4)	29.3 (19.4-39.3)	92.1 *** (86.7-97.5)	38.9 (25.6-52.1)	16.2 (5.6-26.8)
School characteristics								
School type								
Private	23.0 (15.5-30.4)	93.7 (90.5-96.9)	23.5 (13.4-33.5)	17.7 (9.3-26.0)	31.6 (23.7-39.4)	95.6 (91.8-99.5)	36.8 (29.0-44.7)	15.5 (8.1-22.9)
Public	26.4 (19.0-33.7)	95.4 (92.6-98.3)	26.1 (21.2-31.1)	17.0 (11.2-22.9)	26.4 (18.3-34.5)	93.8 (89.6-98.1)	37.8 (29.6-46.0)	16.2 (9.9-22.5)
Study period								
Day	23.7 (18.1-29.3)	94.3 (91.9-96.7)	24.4 (17.5-31.3)	17.6 (11.5-23.6)	30.1 (23.8-36.5)	95.3 (92.3-98.4)	38.5 * (32.0-45.0)	15.3 (9.6-21.0)
Night	31.1 (14.3-47.8)	95.7 (89.6-100.0)	25.5 (8.5-42.6)	15.9 (7.4-24.4)	25.3 (14.1-36.6)	92.1 (83.6-100.0)	28.2 ** (19.4-37.0)	19.0 (9.3-28.7)
Childhood abuse								
Emotional								
No	14.1 * (5.7-22.6)	90.0 (80.0-100.0)	7.3 ^#^ (0.0-15.1)	11.2 (3.7-18.7)	18.4 (6.3-30.6)	90.9 *** (84.4-97.4)	26.9 (14.8-39.1)	5.1 * (0.0-11.5)
Yes	26.3 * (20.0-32.6)	95.2 (92.9-97.6)	27.9 ^#^ (20.5-35.3)	18.7 (12.0-25.3)	32.8 (25.3-40.3)	95.9 *** (93.2-98.7)	39.7 (32.8-46.7)	19.2 * (13.0-25.4)
Physical								
No	20.3 (13.0-27.6)	93.5 (90.2-96.9)	20.3 (11.9-28.7)	13.9 (8.4-19.3)	29.3 (11.1-33.6)	92.5 * (87.4-97.6)	30.4 (17.7-43.1)	13.3 (6.4-20.2)
Yes	27.8 (20.5-35.2)	95.2 (91.5-98.8)	28.3 (20.9-35.7)	21.1 (12.1-30.1)	34.1 (27.6-40.6)	97.2 * (94.8-99.7)	42.2 (35.1-49.3)	16.3 (9.6-22.9)
Sexual								
No	23.7 (18.1-29.4)	93.9 (91.5-96.2)	23.0 (16.7-29.4)	14.8 *** (9.9-19.7)	28.1 (21.7-34.5)	94.5 (91.5-97.6)	36.9 (30.9-42.9)	15.7 (10.3-21.1)
Yes	29.4 (9.7-49.1)	97.2 (91.5-100.0)	35.1 (15.1-55.2)	29.8 *** (9.2-50.3)	49.9 (20.4-79.5)	100.0 (-)	41.4 (10.6-72.1)	16.4 (0.0-38.0)
Emotional neglect								
No	21.7 (11.5-32.0)	89.7 *** (81.7-97.7)	16.4 (6.2-26.6)	9.2 *** (1.8-16.5)	15.4 *** (1.8-28.9)	92.5 (86.5-98.5)	29.7 (14.2-45.2)	11.3 (1.3-21.4)
Yes	25.1 (19.3-30.8)	95.5 *** (93.4-97.6)	26.6 (19.3-33.9)	19.0 *** (12.4-25.6)	33.4 *** (27.1-39.7)	95.8 (92.9-98.7)	39.7 (33.2-46.2)	16.9 (10.9-22.9)
Physical neglect								
No	21.9 (15.8-28.1)	94.7 (91.1-98.2)	20.8 *** (12.9-28.7)	13.4 * (8.4-18.5)	21.5 * (13.7-29.4)	95.3 (91.1-99.6)	31.7 *** (24.4-39.0)	7.9 ^#^ (2.3-13.5)
Yes	27.9 (19.8-36.0)	93.7 (89.2-98.1)	29.0 *** (23.0-35.0)	23.5 * (14.5-32.5)	38.1 * (28.7-47.5)	94.9 (90.6-99.2)	43.9 *** (34.8-53.1)	24.1 ^#^ (15.3-32.8)
Community violence and alcohol consumption								
Community violence								
No	16.1 ^#^ (8.7-23.5)	95.7 (92.5-99.0)	18.7 *** (11.3-26.2)	12.9 (6.7-19.0)	20.6 * (11.7-29.5)	93.5 (88.4-98.6)	30.2 *** (21.4-39.0)	11.6 *** (5.6-17.5)
Yes	31.4 ^#^ (24.3-35.8)	93.1 (88.7-97.6)	28.8 *** (20.2-37.4)	20.1 (11.4-28.7)	35.3 * (27.3-43.3)	95.6 (92.4-98.9)	41.4 *** (34.0-48.7)	17.9 *** (11.4-24.3)
Has friends that commited criminal acts								
No	21.4 ^#^ (16.4-26.4)	93.2 (90.4-96.1)	17.1 ^#^ (10.4-23.8)	15.3 (9.6-21.0)	20.0 ^#^ (13.0-26.9)	94.0 (90.0-98.1)	24.6 ^#^ (15.7-33.5)	13.5 (7.3-19.8)
Yes	31.9 ^#^ (22.4-38.5)	97.8 (94.7-100.0)	42.2 ^#^ (33.7-50.8)	21.9 (11.3-32.4)	41.4 ^#^ (32.3-50.5)	95.9 (92.5-99.2)	53.0 ^#^ (45.1-60.9)	18.8 (11.8-25.8)
Alcohol consumption in the last three months								
Never	25.1 (13.5-36.6)	90.8 (84.6-97.1)	21.6 *** (13.2-30.0)	20.4 (9.6-31.3)	20.6 * (7.8-33.5)	89.8 (82.0-97.5)	33.6 * (20.2-47.2)	14.6 *** (5.1-24.1)
Sometimes	21.5 (14.5-28.4)	95.2 (91.9-98.4)	21.2 *** (11.7-30.7)	16.9 (8.9-24.9)	23.4 * (17.0-29.8)	95.6 (90.6-100.0)	28.1 * (19.8-36.4)	14.2 *** (8.0-20.4)
Often	30.3 (21.2-39.4)	96.1 (91.9-98.4)	31.4 *** (22.8-42.0)	16.0 (8.7-23.3)	43.8 * (29.8-57.8)	97.5 (93.7-100.0)	50.9 * (39.4-62.4)	18.7 *** (8.7-28.6)

95%CI: 95% confidence interval.

Note: all estimates considered sample weights.

* p-value < 0.05;

** Due to the small number of individuals who declared themselves as spiritualists and/or followers of African-based religions (*umbanda* or *candomblé*), these categories were added to the “Others” category;

*** p-value < 0.10;

^#^ p-value < 0.001.

Table 4 presents the perpetration profiles. Prevalence of physical violence did not differ between demographic, socioeconomic, family, and school characteristic subgroups, but threatening behavior was reported more frequently among girls attending night classes. Emotional violence was more frequent among girls in the upper economic classes and boys who studied in private schools and attended daytime classes. Prevalence of sexual abuse perpetration was almost four times higher among girls who were victims of childhood sexual abuse. This picture differed among boys, but those who suffered emotional and physical abuse and emotional neglect as children reported perpetrating threatening acts more often. Those who suffered childhood emotional abuse perpetrated more than two- and three-folds physical and sexual dating violence, respectively. Girls and boys who had friends involved in youth violence also showed a higher prevalence of physical violence perpetration. Threatening behavior and sexual violence perpetration were also more frequent among this subgroup in boys. Notably, girls who consumed alcohol more frequently threatened their boyfriends. Among boys, those who consumed alcohol frequently perpetrated more threats, and emotional and physical violence against their partners.

**Table 4:** Prevalence of dating violence perpetration according to subgroups among adolescent students from Rio de Janeiro, Brazil.

Variables	Threatening behavior	Emotional violence	Physical violence	Sexual violence	Threatening behavior	Emotional violence	Physical violence	Sexual violence
% (95%CI)	% (95%CI)	% (95%CI)	% (95%CI)	% (95%CI)	% (95%CI)	% (95%CI)	% (95%CI)
Demographic profile								
Age (years)								
15 ≤ 16	28.7 (16.9-40.5)	98.6 (96.4-100.0)	38.0 (24.2-51.8)	7.4 * (2.9-11.9)	18.4 (10.8-25.9)	92.8 (86.8-98.8)	22.5 (14.9-30.0)	10.8 (3.4-18.3)
17	34.3 (25.6-42.9)	94.8 (90.9-98-8)	33.8 (25.3-42.4)	3.1 * (0.2-6.6)	20.5 (9.3-31.7)	91.7 (86.5-96.8)	19.2 (9.6-28.7)	14.9 (6.3-23.6)
≥ 18-19	39.5 (24.9-54.2)	93.5 (87.3-99.6)	42.8 (29.4-56.2)	11.8 * (3.5-20.1)	26.7 (16.3-37.0)	92.1 (87.3-96.7)	20.1 (11.0-29.2)	16.4 (9.5-23.3)
Current religion								
Catholic	33.8 (21.6-46.0)	97.2 (94.0-100.0)	38.8 (22.6-54.9)	9.8 (4.5-15.1)	16.9 (5.9-27.9)	92.4 (86.2-98.6)	21.7 (10.5-32.9)	16.1 (5.9-26.2)
Evangelical	40.4 (28.0-52.7)	98.3 (95.9-100.0)	46.2 (33.7-58.7)	9.8 (2.3-17.1)	29.7 (18.2-41.3)	91.5 (85.8-97.1)	25.1 (11.4-38.7)	12.6 (4.0-21.2)
Others **	31.0 (20.0-42.0)	92.3 (85.2-99.3)	28.7 (15.0-42.3)	4.4 (0.0-9.1)	14.9 (0.0-33.1)	90.3 (79.6-100.0)	18.9 (0.0-38.3)	10.1 (0.0-24.7)
Without religion	25.7 (13.0-38.5)	97.6 (93.9-100.0)	36.2 (22.2-50.1)	3.9 (0.0-8.4)	21.2 (12.2-30.3)	92.9 (87.4-98.4)	17.0 (9.3-24.6)	13.3 (6.7-20.0)
Socioeconomic status								
Economic status								
A-B (wealthier)	31.6 (22.9-40.3)	98.7 *** (97.0-100.0)	37.0 (25.6-48.4)	5.9 (2.6-9.1)	22.0 (15.3-28.7)	94.6 (91.1-98.1)	20.4 (14.4-26.4)	12.7 (8.0-17.5)
C-D-E (poorer)	31.7 (21.8-41.6)	93.2 *** (88.5-97.9)	36.3 (25.1-47.4)	6.5 (2.2-10.8)	25.0 (14.4-35.5)	89.7 (82.3-97.1)	23.7 (12.4-34.9)	12.7 (4.0-21.2)
Maternal education (years of schooling)								
≤ 5	35.6 (25.5-45.7)	97.1 (94.4-99.8)	40.2 (29.7-50.7)	4.8 (1.4-8.1)	24.3 (18.1-30.5)	93.1 (89.4-96.8)	17.8 (11.2-24.3)	13.6 (6.5-20.7)
6-8	27.6 (17.3-37.9)	96.8 (93.6-100.0)	32.3 (17.1-47.5)	6.6 (1.3-11.8)	18.2 (9.0-27.4)	95.1 (90.3-99.8)	19.3 (10.6-28.4)	11.4 (5.1-17.6)
> 8	34.7 (19.8-49.6)	93.0 (85.2-100.0)	39.5 (24.0-55.0)	13.7 (2.4-25.0)	28.0 (15.6-40.4)	82.3 (67.6-97.0)	30.6 (16.9-44.4)	20.6 (6.9-34.3)
Family structure								
Live with both parents								
No	35.1 (27.8-42.4)	95.9 (93.1-98.7)	37.8 (27.1-48.5)	7.4 (2.5-12.3)	20.1 (12.9-27.1)	92.4 (87.8-96.9)	18.7 (12.0-25.4)	12.7 (8.0-17.3)
Yes	29.0 (17.1-41.0)	97.2 (94.3-100.0)	37.4 (26.4-48.3)	6.3 (2.7-9.9)	23.4 (15.7-31.0)	92.0 (87.2-96.8)	23.9 (11.8-36.0)	15.2 (7.0-23.3)
School characteristics								
School type								
Private	28.1 (17.4-38.7)	97.8 (95.5-100.0)	34.7 (21.5-47.9)	5,0 (1.4-8.6)	19.3 (13.2-25.3)	96.5 ^#^ (92.8-100.0)	19.0 (11.0-26.9)	11.7 (6.9-16.5)
Public	39.1 (30.6-47.7)	94.5 (91.4-91.4)	42.1 (34.9-49.2)	9.8 (4.7-14.9)	24.6 (17.8-31.4)	85.9 ^#^ (79.4-92.4)	23.4 (16.2-30.6)	16.6 (10.1-23.1)
Study period								
Day	30.6 *** (22.7-38.4)	96.4 (94.4-98.4)	37.0 (27.6-46.3)	6.3 (3.4-9.2)	20.6 (15.8-25.3)	93.9 ^#^ (90.3-97.6)	21.0 (14.9-27.1)	12.8 (8.6-17.0)
Night	51.8 *** (34.4-69.3)	97.8 (93.4-100.0)	43.7 (25.4-62.0)	12.9 (0.4-25.7)	27.3 (14.2-40.5)	80.2 ^#^ (67.7-92.7)	18.9 (5.3-32.6)	19.9 (9.7-30.2)
Childhood abuse								
Emotional								
No	26.9 (14.5-39.2)	95.4 (88.7-100.0)	27.7 (12.0-43.4)	5.9 (90.8-11.0)	8.8 *** (1.2-16.5)	87.6 (80.8-94.3)	10.1 ^#^ (3.6-16.7)	3.8 ^#^ (0.0-7.9)
Yes	33.4 (23.8-43.0)	96.7 (94.8-98.6)	39.5 (29.0-50.0)	7.0 (3.6-10.5)	24.9 *** (18.5-31.1)	93.4 (89.2-97.6)	24.0 ^#^ (16.7-31.3)	17.1 ^#^ (12.2-22.0)
Physical								
No	30.6 (21.9-39.3)	96.2 (92.9-99.5)	32.8 (23.5-42.1)	4.5 * (1.9-7.1)	12.6 *** (5.3-19.8)	89.0 (83.8-94.2)	15.0 * (6.5-23.6)	12.6 (5.5-19.6)
Yes	34.0 (24.7-43.2)	96.8 (94.4-99.0)	42.2 (30.2-54.2)	9.2 * (3.8-14.6)	26.8 *** (20.1-33.5)	94.0 (89.7-98.2)	24.7 * (18.1-31.4)	14.7 (9.0-20.5)
Sexual								
No	32.6 (25.4-39.3)	95.9 (93.7-98.0)	37.8 (30.0-46.0)	4.9 ^#^ (2.7-7.0)	21.4 (16.6-26.3)	91.7 (88.1-94.6)	21.1 (14.9-27.1)	13.1 (8.7-17.5)
Yes	29.6 (9.3-49.9)	100.0 (-)	36.9 (15.2-58.5)	17.6 ^#^ (3.8-31.3)	20.2 (0.0-43.0)	100.0 (-)	16.3 (0.0-38.8)	22.4 (0.0-46.9)
Emotional neglect								
No	31.2 (19.2-43.2)	96.3 (92.1-100.0)	37.3 (21.0-53.5)	3.1 (0.0-7.6)	6.2 ^#^ (0.3-12.0)	88.3 (80.6-96.0)	16.5 (13.3-31.7)	2.6 ^#^ (0.0-6.4)
Yes	32.8 (24.7-40.9)	96.6 (94.4-98.7)	37.8 (29.0-46.7)	7.8 (4.4-11.2)	25.3 ^#^ (20.0-30.7)	93.5 (89.8-97.1)	22.2 (16.0-28.4)	16.8 ^#^ (12.3-21.3)
Physical neglect								
No	29.2 * (21.6-36.7)	96.6 (93.9-99.3)	33.0 (23.6-42.4)	6.2 (3.3-9.0)	17.8 (11.6-23.9)	91.8 (86.6-96.9)	16.4 (9.0-23.8)	8.8 (2.4-15.3)
Yes	37.4 * (27.3-47.5)	96.2 (93.1-99.3)	43.9 (30.6-57.2)	8.1 (3.0-13.3)	25.2 (18.6-32.0)	93.0 (88.0-98.1)	26.0 (17.1-34.9)	19.3 (13.3-25.2)
Community violence and alcohol consumption								
Community violence								
No	27.0 *** (18.3-35.7)	97.3 (94.3-100.0)	32.2 (21.7-42.6)	6.2 (1.6-10.8)	19.8 (11.4-28.0)	90.7 (85.4-96.1)	20.5 (9.4-31.5)	10.5 (3.9-17.0)
Yes	36.8 *** (27.6-45.9)	95.8 (93.2-98.4)	41.8 (30.0-53.6)	6.1 (2.4-9.7)	22.6 (16.6-28.7)	93.5 (89.9-95.4)	21.3 (14.8-27.8)	15.1 (9.7-20.4)
Has friends that commited criminal acts								
No	29.3 (20.1-38.4)	95.7 * (93.0-98.4)	31.0 *** (21.5-40.5)	5.2 (2.7-7.6)	13.8 ^#^ (8.9-18.7)	91.3 (87.1-95.4)	14.6 *** (6.3-22.8)	8.8 *** (3.9-13.7)
Yes	39.5 (29.1-50.0)	99.2 * (97.8-100.0)	53.5 *** (39.3-67.7)	10.1 (2.3-17.8)	30.7 ^#^ (22.5-38.9)	94.3 (90.2-98.5)	28.5 *** (20.2-36.8)	19.8 *** (11.8-27.8)
Alcohol consumption in the last three months								
Never	27.6 *** (16.3-38.9)	95.8 (91.3-100.0)	36.6 * (21.9-51.4)	5.2 (2.7-7.6)	12.8 ^#^ (4.0-21.6)	86.3 *** (79.1-93.5)	13.7 *** (7.1-24.7)	8.4 (0.9-15.8)
Sometimes	29.6 *** (21.1-38.0)	96.5 (93.4-99.6)	32.3 * (22.3-42.4)	5.2 (26.8-76.5)	15.7 ^#^ (9.9-21.5)	91.8 *** (85.5-98.2)	15.7 *** (7.9-23.5)	13.5 (7.6-19.4)
Often	44.2 *** (33.9-54.5)	97.8 (94.7-100.0)	51.0 * (34.7-67.3)	11.3 (3.3-19.3)	34.4 ^#^ (23.1-45.7)	96.8 *** (93.8-99.7)	31.5 *** (19.9-43.0)	17.8 (8.8-26.8)

95%CI: 95% confidence interval.

Note: all values include sample weights.

* p-value < 0.10;

** Due to the small number of individuals who declared themselves as spiritualists and/or followers of African-based religions (*umbanda* or *candomblé*), these categories were added to the “Others” category;

*** p-value < 0.05;

^#^ p-value < 0.001.

## Discussion

Prevalence of different forms of dating violence among adolescent students is high and conspicuous, calling for preventive intervention strategies. Similar prevalence among boys and girls cast some doubt on the idea of their fixed roles as perpetrators and victims, respectively. Nonetheless, we found some specificities. As in previous studies, girls reported perpetrating threatening behaviors and physical violence more often than boys, whereas boys recognized practicing sexual violence more often ^2^. While present in all subgroups, dating violence distribution was not homogeneous. Overall, those who were victims of child abuse, lived in more violent areas of the city, had friends that committed criminal acts, and consumed alcohol more frequently were more vulnerable to dating violence, whether as victims or perpetrators.

Emotional abuse was the most frequent form of violence. Although not studied as often as physical or sexual assault, this type of dating violence appears to be the most common in other countries as well ^13^. A systematic review conducted by Espelage et al. ^35^ including European and North American studies drew attention to its reciprocal nature and the need for considering the psychosocial characteristics of adolescents to better understand why emotional abuse is so common during this life period. According to the authors, the need for self-assertion among peers can encourage acts of emotional violence to exhibit power and control over one’s partner. Social skills, like those needed for solving conflicts through healthy negotiation, may not be fully developed yet. In this scenario, acts such as inciting jealousy or showing hostility towards a partner are naturalized because they seem the only resource in the face of disagreements. Regardless, note that in our survey participants who answered positively at least one of the subscale items were considered positive for that form of violence. This methodological choice may have increased sensitivity over specificity, possibly overestimating dating violence prevalence and especially emotional abuse since this subscale has twice as many component items as the others.

Like other surveys ^2^, our results identified that girls reported perpetrating physical violence more often than boys. Among adults, the role of women as assault perpetrators against partners has been debated for a long time ^36^. Some authors focusing on heterosexual relationships argue that female engagement in intimate partner violence is motivated by resistance and reaction to a male’s attempts at power and control ^37^, especially in patriarchal societies ^38^. Other researchers soften the rigid roles of men and women in intimate partner violence, reinforcing the importance of considering intimate partner dynamics in these situations ^36^.

Higher prevalence of physical dating violence perpetration among girls may be explained by considering that most of these situations involve minor, less harmful acts rather than the extreme behaviors that can even lead to death more often perpetrated by men. High prevalence of boys reporting physical victimization reinforces the role girls can have as perpetrators in heterosexual relationships. Yet, this finding was unexpected given the results from a qualitative research conducted in the same schools. According to Costa et al. ^39^, boys recognizing themselves as victims was quite rare since they saw themselves as stronger and thus capable of stopping aggressive acts from their partners. Perhaps using a validated questionnaire comprising different acts of physical violence rather than direct questions helped to uncover these situations in our study.

Despite the lower rate of sexual abuse, nearly one in five students reported having been a victim and almost 10% stated having committed acts of this nature in their intimate relationships. The seriousness of sexual violence calls for more discussions about consent and respect in romantic and sexual relationships in society, particularly in schools where social networks usually occur during adolescence ^40^. Interventions aiming to promote healthy and egalitarian dating relationships should emphasize that mutual agreement must not be restricted to sexual relations involving penetration but extended to negotiating all forms of sexual interaction.

Unlike previous studies, we found almost no differences in reported victimization of sexual dating violence between boys and girls. Further research on the prevalence of separate item measures could help understand this finding. As a reminder, the sexual abuse subscale comprises only three items: “touched me sexually when I did not want to”; “forced me to have sex when I did not want to”; and “threatened me in an attempt to have sex”. Notably, the first item showed the highest agreement rate among girls (15.8%) and boys (14.7%). The other two acquired less than 6% and, though more common among girls, were insufficient to influence the summary estimate. Looking at the content of these items, we note that the first refers to a “less severe” sexual coercive act whereas the other two depict more complicated situations, including attempted or even accomplished rape. These findings may not allow us to draw a more forceful conclusion, but we can posit that the absence of gender differences in reported victimization resulted from a scalability gap arising from the lack of items capturing intermediate, moderate intensity situations. Since these experiences tend to be more frequent among girls, as pointed out in the literature ^16^, values would have been likely higher than among boys if they were present.

Bronfenbrenner’s ^41^ ecological model can help support the debate on the differences in prevalence among subgroups as this theoretical framework has been widely used to explain the greater vulnerability of certain groups to intimate partner violence and other types of interpersonal violence ^15^ ^,^ ^19^. According to the model, violence results from the interaction between macro-structural, community, relational, and individual risk and protective factors. These dimensions involve cultural, economic, and social characteristics; aspects related to the community where individuals live, work, or study; strategies used to solve parent-child, peer and partner conflict; and characteristics of the involved individuals such as age, gender, temperament and alcohol abuse ^15^ ^,^ ^42^.

Although considered a useful theoretical model to explain violence occurrence, positing that these different spheres have distinct relevance when it occurs during adolescence is still possible. Rather than using individual characteristics to explain the higher level of dating violence in certain subgroups, some authors highlight relational characteristics, especially those involving parent-child interactions, specific contexts of adolescent socialization, and aspects of the romantic relationship itself ^36^. Our results somewhat support this proposition by indicating that dating violence was more frequent among adolescents with a family history of childhood abuse, instigating a discussion on the importance of family bonding in building affective relationships during adolescence. However, the specificities of violence profiles according to gender and alcohol consumption highlight that those individual characteristics are also relevant. The community’s role as a backdrop is also notable when considering the higher prevalence of violence among students living in areas with a high degree of community violence. These findings reinforce the multidimensionality and complexity of the issue and the need for integrated interventions aimed at dating violence prevention and earlier detection of recurring cases.

As presented in the *Results* section, this study identified few statistically significant differences in the prevalence of dating violence victimization between demographic and socioeconomic groups. In isolation, these characteristics may thus not be helpful in determining vulnerable groups for prioritizing and planning interventions. As detailed when addressing the study limitations, the absence of statistically significant differences may be explained by the small sample size within specific subgroups. Regardless, the higher victimization prevalence among adolescents reporting a history of emotional and physical abuse and neglect during childhood is noteworthy. Such a profile reinforces the need to consider adolescents’ relationships within their original families when planning actions aimed at early detection of most vulnerable victims. In this regard, knowledge of child abuse and neglect stories may help unveil already established dating violence situations. Likewise, the need to incorporate community characteristics and adolescent networks into the discussion is evinced when considering that dating violence victimization is more common among adolescents who live in violent neighborhoods and have friends who committed crimes. Violence prevalence among boys who often consume alcohol and the harmful effects this may have during this life cycle suggest that strategies aimed at mitigating dating violence should also encompass actions for preventing alcohol abuse ^13^ ^,^ ^14^ ^,^ ^43^.

The perpetration profile also deserves discussion. Regarding history of family child abuse among adolescent girls, only those victims of sexual abuse reported higher perpetration of sexual violence against their partners. Among boys, however, perpetration of different dating violence types was more frequent among those reporting different forms of child abuse and neglect. Aggressive traits, adjustment difficulties, or low self-esteem are some of the wounds resulting from childhood abuse that can act as mediators between childhood experiences and externalizing behaviors, such as aggression, in adolescence ^44^ ^,^ ^45^. Considering these findings, when identifying adolescents who perpetrate dating violence we should be aware that they may also have been past victims of violence. This would help incorporate strategies aimed at detecting and treating existing mental health issues arising from these adverse childhood experiences, in addition to those aimed at stopping current intimate partner violence. The higher prevalence of dating violence, especially among boys who consume alcohol, also stresses the need to incorporate actions for alcohol abuse prevention in interventions aimed at reducing dating violence ^14^.

All findings must be interpreted in light of some study limitations. Firstly, the wide confidence intervals of the prevalences due to data sparseness within specific subgroups, an issue particularly noticeable regarding dating violence among childhood sexual abuse victims. Point estimates must thus be viewed with some caution in these situations. Similarly, the relatively small sample size in certain subgroups may have led to non-statistically significant prevalence differences. Future research employing larger sample sizes could shed some light on these matters. Another drawback is that adolescent victims or perpetrators of severe dating violence may have dropped out and would thus not have been sampled. Such selectivity may have lessened all forms of violence prevalence estimates, ultimately restricting the possibility of generalizing findings to only those adolescents regularly attending high school. A loss of students from less privileged economic classes must also be acknowledged, as many could have already left school to find a job to supplement family income. Reduced representation of lower socioeconomic classes in the sample - which led us to aggregate families from classes C, D, and E into a single category for subgroup analysis - could result from this. Indigenous and Asian adolescents were also underrepresented and had to be excluded. Future studies should employ sample strategies that oversample these subgroups to allow dating violence estimation among them. Another possible drawback related to school surveys concerns underestimating sensitive topics like the various forms of violence studied. Adolescents can feel embarrassed or insecure in disclosing situations that potentially imply negative institutional ramifications; however, using a self-administered, well-adapted questionnaire to capture violence may have mitigated this shortcoming. Although self-reporting may have failed to identify the perpetration of more severe acts for fear of legal repercussions, this approach seems to be the best option to estimate the prevalence of more subtle forms of violence which usually go unreported to the protection, police, and justice services. Absence of information on sexual orientation and gender identity hindered exploring dating violence patterns among these minorities. Finally, an important study limitation lies in its geographical scope. The research was conducted in a specific area of Rio de Janeiro and may not fully represent the entire municipality. While the participating schools included students from over 30 different neighborhoods, caution is needed when extrapolating prevalence estimates to the broader urban context.

## Conclusions

Our results reinforce the need for public policy interventions to reduce adolescent dating violence in Rio de Janeiro. Although boys and girls are equally victims and perpetrators of different forms of violence, the characteristics that make them more or less vulnerable differ. Hence, each subgroup’s specificities should be considered when planning effective interventions aimed at dating violence prevention. The findings also point to no predetermined victim and perpetrator role in adolescent love relations. Many situations are reciprocal, which certainly amplifies their negative consequences and hinders their full growth and development. However, we cannot ignore that most fatal victims of dating violence are female.

Moreover, the high frequencies of exposure to different types of violence during childhood and the great prevalence of students who declared having seen a murdered body or had a family member killed suggest the transversality of situations in socialization spaces. This sum of adversities implies that any action to address interpersonal violence requires a multidimensional approach and must involve integrated and networked institutions. Schools can play an essential role in this regard due to their symbolic value and important social spaces for children and adolescents. In this perspective, the entire school cycle should be seen as a window of opportunity to address alternative positive parenting strategies to avoid parent-child abuse, to support healthy intimate partner relations, and to discuss the negative consequences of interpersonal violence on adolescent wellbeing.

## References

[1] U.S. Centers for Disease Control and Prevention About teen dating violence..

[2] Wincentak K, Connolly J, Card N (2017). Teen dating violence a meta-analytic review of prevalence rates. Psychol Violence.

[3] Galende N, Ozamiz-Etxebarria N, Jaureguizar J, Redondo I (2020). Cyber dating violence prevention programs in universal populations a systematic review. Psychol Res Behav Manag.

[4] Taquette SR, Monteiro DLM (2019). Causes and consequences of adolescent dating violence a systematic review. J Inj Violence Res.

[5] Rubio-Garay F, López-González MA, Carrasco MA, Amor PJ (2017). The prevalence of dating violence a systematic review. Papeles del Psicólogo.

[6] Quinones C, Navarro A (2022). A 10 year (2011-2021) systematic review of teen dating violence prevention programs. J Inj Violence Res.

[7] Tomaszewska T, Schuster I (2021). Prevalence of teen dating violence in Europe a systematic review of studies since 2010. New Dir Child Adolesc Dev.

[8] Oliveira QBM, Assis SG, Njaine K, Pires TO (2014). Namoro na adolescência no Brasil circularidade da violência psicológica nos diferentes contextos relacionais. Ciênc Saúde Colet.

[9] Minayo MCS, Assis SG, Njaine K (2011). Amor e violência: um paradoxo das relações de namoro e do 'ficar' entre jovens brasileiros.

[10] Beserra MA, Leitão MNC, Fernandes MID, Scatena L, Vidinha TSS, Silva LMP (2015). Prevalence of dating violence among adolescents from Brazilian public schools of Recife/PE - Brazil. Referência - Revista de Enfermagem.

[11] Borges JL, Giordani JP, Wendt B, Trentini CM, Dell'Aglio DD (2020). Patterns of perpetration and perceptions of teend ating violence.. Psico USF.

[12] Sandoval GA, Marinho F, Delaney R, Pinto IV, Lima CMD, Costa RM (2020). Mortality risk among women exposed to violence in Brazil a population-based exploratory analysis. Public Health.

[13] Bowen E, Walker K, Bowen E, Walker K (2015). The psychology of violence in adolescent romantic relationships.

[14] Haynes EE, Strauss CV, Stuart GL, Shorey RC (2017). Drinking motives as a moderator of the relationship between dating violence victimization and alcohol problems. Violence Against Women.

[15] Krug GE, Dahlberg LL, Mercy AJ, Zwi BA, Lozano R (2002). World report on violence and health.

[16] Barreira AK, Lima ML, Bigras M, Njaine K, Assis SG (2014). Directionality of physical and psychological dating violence among adolescents in Recife, Brazil. Rev Bras Epidemiol.

[17] Veríssimo AVR, Silva EA, Soares KHD, Amaral ELS, Brandão W, Ludermir AB (2022). Prevalência e fatores associados à violência no namoro entre adolescentes de escola pública.. Rev Gaúch Enferm.

[18] Borges JL, Dell'Aglio DD (2020). Esquemas iniciais desadaptativos como mediadores entre os maus tratos na infância e a violência no namoro na adolescência.. Ciênc Saúde Colet.

[19] Rothman EF, Wolfe DA, Temple JR (2018). Adolescent dating violence: theory, research, and prevention.

[20] Reed LA, Tolman RM, Ward LM (2017). Gender matters experiences and consequences of digital dating abuse victimization in adolescent dating relationships. J Adolesc.

[21] Yu R, Pepler DJ, van de Bongardt D, Josephson WL, Connolly J (2018). Internalizing symptoms and dating violence perpetration in adolescence.. J Adolesc.

[22] DATA.RIO População Residente e Estimada - Brasil, Estado do Rio de Janeiro e Município do Rio de Janeiro e Regiões Administrativas (RA) - 2000/2010/2013-2016/2020..

[23] DATA.RIO Índice de Desenvolvimento Social (IDS) por Áreas de Planejamento (AP), Regiões de Planejamento (RP), Regiões Administrativas (RA), Bairros e Favelas do Município do Rio de Janeiro - 2010..

[24] Prefeitura do Rio de Janeiro Informações sobre a cidade do Rio de Janeiro..

[25] Wolfe DA, Scott K, Reitzel-Jaffe D, Wekerle C, Grasley C, Straatman AL (2001). Development and validation of the Conflict in Adolescent Dating Relationships Inventory. Psychol Assess.

[26] Hokoda A, Ramos-Lira L, Celaya P, Vilhauer K, Angeles M, Ruiz S (2006). Reliability of translated measures assessing dating violence among Mexican adolescents. Violence Vict.

[27] Jouriles EN, McDonald R, Garrido E, Rosenfield D, Brown AS (2005). Assessing aggression in adolescent romantic relationships can we do it better?. Psychol Assess.

[28] Jouriles EN, Garrido E, Rosenfield D, McDonald R (2009). Experiences of psychological and physical aggression in adolescent romantic relationships links to psychological distress. Child Abuse Negl.

[29] Reichenheim ME, Marques ES, Moraes CL (2022). Structural validity of the Conflict in Adolescent Dating Relationships Inventory (CADRI) a reduced version for use on respondents as victims and perpetrators. Child Abuse Negl.

[30] Associação Brasileira de Empresas de Pesquisa. Critério de Classificação Econômica Brasil.

[31] Grassi-Oliveira R, Cogo-Moreira H, Salum GA, Brietzke E, Viola TW, Manfro GG (2014). Childhood Trauma Questionnaire (CTQ) in Brazilian samples of different age groups findings from confirmatory factor analysis. PLoS One.

[32] Bernstein DP, Fink L (1998). Childhood Trauma Questionnaire: a retrospective self-report - manual.

[33] Centro de Estudo de Segurança e Cidadania (2012). Questionário JUVIPOL - Juventude, Violência e Polícia.

[34] Henrique IFS, Micheli D, Lacerda RB, Lacerda LA, Formigoni MLOS (2004). Validação da versão brasileira do teste de triagem do envolvimento com álcool, cigarro e outras substâncias (ASSIST). Rev Assoc Med Bras.

[35] Espelage DL, Davis JP, Basile KC, Rostad WL, Leemis RW (2018). Alcohol, prescription drug misuse, sexual violence, and dating violence among high school youth. J Adolesc Health.

[36] Straus M (2010). Thirty years of denying the evidence on gender symmetry in partner violence implications for prevention and treatment. Partner Abuse.

[37] Heise L, Ellsberg M, Gottmoeller M (2002). A global overview of gender-based violence. Int J Gynaecol Obstet.

[38] Hanser RD, Jackson NA (2007). Encyclopedia of domestic violence.

[39] Costa SF, Taquette SR, Moraes CL, Souza LMBM, Moura MP (2020). Contradições acerca da violência sexual na percepção de adolescentes e sua desconexão da lei que tipifica o "estupro de vulnerável".. Cad Saúde Pública.

[40] Joppa MC (2020). Dating violence in adolescence implications for girls' sexual health. J Pediatr Adolesc Gynecol.

[41] Bronfenbrenner U (1979). Contexts of child rearing. Am Psychol.

[42] World Health Organization (2019). RESPECT women: preventing violence against women.

[43] Choi HJ, Elmquist J, Shorey RC, Rothman EF, Stuart GL, Temple JR (2017). Stability of alcohol use and teen dating violence for female youth A latent transition analysis. Drug Alcohol Rev.

[44] Ferreira MDF, Moraes CL, Braga JU, Reichenheim ME, Veiga GVD (2018). Abusive alcohol consumption among adolescents a predictive model for maximizing early detection and responses. Public Health.

[45] Sideli L, Murray RM, Schimmenti A, Corso M, La Barbera D, Trotta A (2020). Childhood adversity and psychosis a systematic review of bio-psycho-social mediators and moderators. Psychol Med.

